# Risk assessment of substance use disorders based on the human leukocyte antigen (HLA)

**DOI:** 10.1038/s41598-023-35305-2

**Published:** 2023-05-26

**Authors:** Lisa M. James, Apostolos P. Georgopoulos

**Affiliations:** 1 The HLA Research Group, Brain Sciences Center (11B), Department of Veterans Affairs Health Care System, Minneapolis VAHCS, One Veterans Drive, Minneapolis, MN 55417 USA; 2 Department of Neuroscience, University of Minnesota Medical School, Minneapolis, MN 55455 USA; 3 Department of Psychiatry, University of Minnesota Medical School, Minneapolis, MN 55455 USA; 4 Department of Neurology, University of Minnesota Medical School, Minneapolis, MN 55455 USA

**Keywords:** Immunogenetics, Addiction

## Abstract

Substance use disorders (SUDs) are common and costly conditions that are partially attributable to genetic factors. In light of immune system influences on neural and behavioral aspects of addiction, the present study evaluated the influence of genes involved in the human immune response, human leukocyte antigen (HLA), on SUDs. We used an immunogenetic epidemiological approach to evaluate associations between the population frequencies of 127 HLA alleles and the population prevalences of six SUDs (alcohol, amphetamine, cannabis, cocaine, opioid, and “other” dependence) in 14 countries of Continental Western Europe to identify immunogenetic profiles of each SUD and evaluate their associations. The findings revealed two primary groupings of SUDs based on their immunogenetic profiles: one group comprised cannabis and cocaine, whereas the other group comprised alcohol, amphetamines, opioids, and “other” dependence. Since each individual possesses 12 HLA alleles, the population HLA-SUD scores were subsequently used to estimate individual risk for each SUD. Overall, the findings highlight similarities and differences in immunogenetic profiles of SUDs that may influence the prevalence and co-occurrence of problematic SUDs and may contribute to assessment of SUD risk of an individual on the basis of their HLA genetic makeup.

## Introduction

Substance use disorders (SUDs) are common worldwide, resulting in significant health and economic costs^1^. It is well established that both genetic and environmental influences shape substance use^2^, with 50–60% of SUD risk attributed to heritable contributions^3^. Although specific genetic influences on alcohol use disorders have been widely investigated and documented, genetic influences on other SUDs such as cocaine, opioids, and cannabis are limited despite their high prevalences, leading researchers to highlight the need for additional research on the genetics of those SUDs^4^. Concurrently, in light of evidence demonstrating immune system influences on neural and behavioral aspects of addiction^5^, there has been increasing emphasis on immunological and psychoneuroimmunological aspects of SUDs^5,6^. Here, we bridge those lines of research by evaluating the immunogenetics of six SUDs—namely, alcohol, amphetamine, cannabis, cocaine, opioid, and “other” dependence (a residual category including hallucinogens, inhalants, sedatives, and solvent dependence)—according to their human leukocyte antigen (HLA) profiles.

The HLA region of chromosome 6 codes for two classical types of cell surface proteins that are instrumental in immune system surveillance and elimination of non-self antigens. Class I HLA molecules (HLA-A, B, C) bind and export small peptides from proteolytically degraded cytosolic foreign antigens to the cell surface for presentation to CD8+ cytotoxic T cells, signaling cell destruction. Class II HLA molecules (HLA-DPB1, DQB1, DRB1) present larger peptides derived from endocytosed exogenous antigens to CD4+ T cells to facilitate B cell mediated antibody production and adaptive immunity. The HLA region is the most highly polymorphic region of the human genome^7^, and variation in HLA has been shown to contribute to variation in disease susceptibility^8^. HLA-disease associations have been most widely established for autoimmune disorders^9^; however, HLA associations have been increasingly documented for diseases not traditionally characterized primarily by immune system dysregulation including various psychiatric conditions^10^. With regard to SUDs, HLA has been implicated as an important genetic factor associated with alcohol dependence^11,12^ and alcohol-related liver disease^13^, although methodological limitations have rendered findings of HLA associations with alcohol dependence across studies largely inconsistent^14^. HLA associations with other SUDs have received modest attention in humans (c.f., ref.^15^); however, major histocompatibility complex class I (MHCI; HLA Class I equivalent) expression in dopaminergic neurons has been shown to play a key role in suppressing reward-seeking behavior related to cocaine use in mice^16^, and morphine administration in rats has been shown to suppress MHCII (HLA Class II equivalent) expression^17^, highlighting the interactions between addictive substances and immunogenetics. Here, we used an immunogenetic epidemiological approach to evaluate associations between the population frequencies of a large number of HLA alleles and the population prevalences of SUDs in Continental Western Europe to begin to elucidate immunogenetic profiles for SUDs (SUD^HLA^). Furthermore, since SUDs frequently co-occur^18^, we evaluated associations between SUD^HLA^ profiles to identify immunogenetic influences underlying their co-occurrence. Finally, since each individual carries 12 HLA alleles (two alleles per HLA gene), we used the population scores to estimate individual SUD risk.

## Results

### Immunogenetics profiles

**Figure 1:**
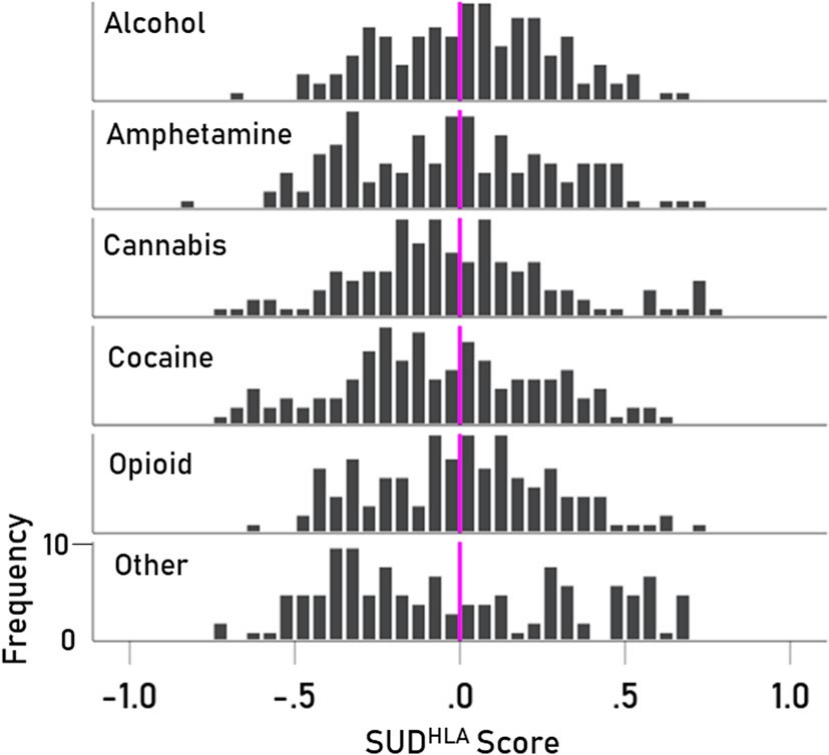
Frequency distributions of immunogenetic SUD^HLA^ scores for the 6 SUDs studied. N = 127 for each distribution.

The immunogenetic scores of the 6 SUDs and the alleles of 6 classical HLA genes A, B, C, DPB1, DQB1, and DRB1 (127 alleles in total) are given in Tables 1, 2, 3, 4, 5, 6, respectively, their frequency distributions are plotted in Fig. 1, and their descriptive statistics are given in Table 7. In the permutation test, where SUD prevalences were randomly paired with HLA allele frequencies, not a single case (out of 1,000,000 runs) was found to match the observed SUD^HLA^ profiles of any of the 6 SUDs, thus rejecting the null hypothesis that the observed profile could be accounted for by chance (P < 1 × 10^–6^). The same results were obtained in the ranks version of the random permutations test, which relaxed the requirement of an exact SUD^HLA^ match and focused instead on a match of the ranked SUD^HLA^ scores: no cases of an exact match was found, thus rejecting again the null hypothesis that the SUD^HLA^ profiles could be accounted for by chance (P < 10^–6^). Therefore, we analyzed the 6 sets of SUD^HLA^ scores with the following results.

**Table 1:** Immunogenetic SUD^HLA^ scores of HLA Class I gene A alleles.

Index	Allele	Alcohol	Amphetamine	Cannabis	Cocaine	Opioid	Other
1	A*01:01	0.0034	− 0.5425	0.1939	0.3163	0.2992	− 0.1076
2	A*02:01	0.2807	0.0030	− 0.1959	− 0.2605	0.6090	0.5082
3	A*02:05	− 0.0630	0.3606	− 0.0645	− 0.0811	− 0.4102	− 0.3106
4	A*03:01	0.2112	0.4785	− 0.0540	0.0096	0.2515	0.6723
5	A*11:01	− 0.2656	− 0.2486	0.1926	0.0861	− 0.1668	− 0.2869
6	A*23:01	0.1245	− 0.3270	0.3106	0.1450	− 0.3169	− 0.2368
7	A*24:02	− 0.0934	0.1738	− 0.6155	− 0.3551	− 0.2419	− 0.1192
8	A*25:01	0.0199	0.1863	0.3041	0.3161	0.1131	0.2900
9	A*26:01	− 0.2177	− 0.4537	− 0.0780	0.2362	0.1125	− 0.2687
10	A*29:01	0.0074	0.1014	− 0.4433	− 0.6554	0.0968	0.2732
11	A*29:02	0.1584	− 0.0107	0.7037	0.3834	− 0.0858	− 0.0740
12	A*30:01	− 0.3037	0.0323	− 0.0812	0.0976	− 0.2227	− 0.2340
13	A*30:02	− 0.2134	− 0.1150	0.5833	0.2144	− 0.4162	− 0.4200
14	A*31:01	0.1285	0.4397	0.2229	0.2837	0.1355	0.5189
15	A*32:01	− 0.0947	− 0.5384	− 0.0078	− 0.1246	− 0.2145	− 0.3933
16	A*33:01	0.1630	0.2329	0.1144	− 0.1633	− 0.3118	− 0.2499
17	A*33:03	− 0.0359	− 0.3236	− 0.6925	− 0.4897	0.1292	− 0.0998
18	A*36:01	0.1528	0.0055	0.0629	− 0.3031	− 0.2696	− 0.1733
19	A*68:01	0.1872	− 0.2342	− 0.1714	− 0.0981	0.1447	0.0552
20	A*68:02	0.4295	0.1303	0.0379	0.0378	0.3130	0.1606

**Table 2:** Immunogenetic SUD^HLA^ scores of HLA Class I gene B alleles.

Index	Allele	Alcohol	Amphetamine	Cannabis	Cocaine	Opioid	Other
1	B*07:02	0.2258	0.3103	0.0899	0.3583	0.0792	0.4926
2	B*08:01	0.2206	− 0.0203	0.2345	0.2998	0.3411	0.3452
3	B*13:02	− 0.2733	− 0.1226	− 0.1908	0.1261	0.2566	0.2696
4	B*14:01	0.3242	0.4330	0.3533	0.0708	− 0.3781	− 0.2600
5	B*14:02	− 0.0180	0.3773	0.0120	− 0.1012	− 0.4317	− 0.4175
6	B*15:01	0.0805	0.2822	0.1520	0.3491	− 0.0319	0.5880
7	B*15:17	0.0298	0.0083	− 0.3286	− 0.4777	0.1229	− 0.3305
8	B*15:18	− 0.0231	− 0.4356	− 0.1156	− 0.6439	0.0111	− 0.2967
9	B*18:01	− 0.2533	− 0.2008	− 0.0988	− 0.2764	0.0384	− 0.3662
10	B*27:02	− 0.1116	− 0.0095	− 0.7099	− 0.6499	0.4013	0.1383
11	B*27:05	0.2111	0.2181	− 0.0536	0.1423	0.4355	0.5630
12	B*35:01	0.0514	0.3048	− 0.5938	− 0.5695	− 0.0203	− 0.2330
13	B*35:02	0.2369	0.1102	− 0.3741	− 0.2214	0.2455	− 0.1783
14	B*35:03	− 0.2145	− 0.2979	− 0.1821	− 0.3591	0.1455	− 0.3575
15	B*35:08	− 0.2264	− 0.3551	− 0.3797	− 0.6082	− 0.0992	− 0.5084
16	B*37:01	− 0.3898	0.4755	0.1240	0.3300	− 0.3353	0.2630
17	B*38:01	0.0695	− 0.1151	− 0.2716	− 0.2433	− 0.0929	− 0.3093
18	B*39:01	− 0.2630	− 0.1007	− 0.5000	− 0.2676	− 0.4802	− 0.3768
19	B*39:06	0.3369	0.0228	− 0.4484	− 0.7070	0.3564	0.0572
20	B*40:01	0.1198	0.4120	0.0085	0.0354	0.1485	0.5898
21	B*40:02	0.2056	− 0.1317	− 0.6394	− 0.4244	0.1860	− 0.0276
22	B*41:01	0.0445	0.2410	− 0.2818	− 0.2623	0.3632	0.0293
23	B*41:02	0.1830	− 0.2577	− 0.3063	− 0.5077	− 0.0838	0.0131
24	B*44:02	0.4068	− 0.3070	0.0917	0.2445	0.2094	0.1051
25	B*44:03	0.0771	− 0.2237	0.7301	0.4188	− 0.1007	− 0.0849
26	B*44:05	− 0.2122	− 0.3763	− 0.5146	− 0.6698	− 0.3427	− 0.4195
27	B*45:01	0.3040	− 0.0837	0.1214	0.0356	− 0.0812	− 0.3151
28	B*47:01	0.2480	− 0.0289	− 0.2876	− 0.5409	0.0487	0.0791
29	B*49:01	− 0.0954	− 0.1428	0.0930	− 0.2580	− 0.3799	− 0.6071
30	B*50:01	0.2209	0.2763	− 0.0744	− 0.1261	− 0.1867	− 0.2245
31	B*51:01	0.0980	− 0.1535	− 0.2630	− 0.4221	− 0.1513	− 0.7010
32	B*52:01	− 0.2208	− 0.2204	0.0322	− 0.0399	0.4583	0.3026
33	B*55:01	− 0.1032	0.0530	0.7259	0.4993	− 0.1012	0.2901
34	B*56:01	0.1719	0.0183	− 0.3781	− 0.3068	0.7000	0.6532
35	B*57:01	− 0.0786	− 0.4932	0.2259	0.1269	− 0.0555	− 0.0270
36	B*58:01	− 0.2420	− 0.2735	− 0.3212	− 0.5941	− 0.0399	− 0.2308

**Table 3:** Immunogenetic SUD^HLA^ scores of HLA Class I gene C alleles.

Index	Allele	Alcohol	Amphetamine	Cannabis	Cocaine	Opioid	Other
1	C*01:02	− 0.1718	0.1226	− 0.0716	− 0.1944	0.6171	0.5781
2	C*03:03	0.1670	0.5141	0.2251	0.0087	0.2887	0.5060
3	C*04:01	− 0.0630	− 0.0178	− 0.3981	− 0.4085	− 0.0698	− 0.3490
4	C*05:01	0.2746	0.1258	0.7541	0.4082	− 0.0761	0.1349
5	C*06:02	0.0745	− 0.3534	− 0.1280	0.1604	− 0.4208	− 0.2306
6	C*07:01	− 0.3483	− 0.3396	− 0.1910	− 0.1605	0.1333	0.0200
7	C*07:02	0.0910	0.4620	0.2553	0.2746	0.1111	0.4968
8	C*07:04	− 0.1578	− 0.8257	− 0.2095	− 0.0827	0.0108	− 0.1045
9	C*12:02	− 0.3878	− 0.4245	− 0.0451	− 0.0555	− 0.1781	− 0.2716
10	C*12:03	− 0.3420	− 0.4394	− 0.1226	0.0938	− 0.3090	− 0.4865
11	C*14:02	− 0.0897	− 0.5363	− 0.1898	− 0.1459	− 0.2496	− 0.5398
12	C*15:02	− 0.0755	0.0141	− 0.1498	0.0153	− 0.4222	− 0.4858
13	C*16:01	0.1050	0.2766	0.7239	0.5544	− 0.3563	− 0.0813

**Table 4:** Immunogenetic SUD^HLA^ scores of HLA Class II gene DPB1 alleles.

Index	Allele	Alcohol	Amphetamine	Cannabis	Cocaine	Opioid	Other
1	DPB1*01:01	0.2617	0.2504	− 0.0620	− 0.2539	0.3926	0.6516
2	DPB1*02:01	− 0.2681	− 0.0993	− 0.1719	0.1650	− 0.4152	− 0.5933
3	DPB1*02:02	0.0074	− 0.0151	0.2329	− 0.0732	− 0.0401	− 0.0295
4	DPB1*03:01	− 0.1043	0.6202	− 0.2646	− 0.2979	− 0.3131	0.0811
5	DPB1*04:01	0.3486	0.0398	− 0.1327	0.0228	0.4419	0.6623
6	DPB1*04:02	0.0388	− 0.3265	− 0.5976	− 0.6034	0.5166	0.3785
7	DPB1*05:01	0.2940	0.2344	0.2596	− 0.1259	0.0728	0.3495
8	DPB1*06:01	0.5167	0.1922	0.4984	0.2810	0.0489	0.1046
9	DPB1*09:01	− 0.1479	0.4159	− 0.0862	0.1718	− 0.2657	− 0.3620
10	DPB1*10:01	− 0.1049	− 0.3125	0.0920	− 0.2301	0.0397	− 0.3769
11	DPB1*11:01	0.0015	0.1333	0.6627	0.6078	0.1169	0.2867
12	DPB1*13:01	− 0.1249	− 0.5799	0.0647	− 0.1589	0.0476	− 0.3753
13	DPB1*14:01	0.0257	− 0.3096	− 0.0917	− 0.1974	0.0745	− 0.4555
14	DPB1*17:01	− 0.0373	0.0110	0.1456	0.1595	− 0.0381	− 0.5289
15	DPB1*19:01	0.0633	0.2044	0.3364	0.0621	− 0.0087	0.2289

**Table 5:** Immunogenetic SUD^HLA^ scores of HLA Class II gene DQB1 alleles.

Index	Allele	Alcohol	Amphetamine	Cannabis	Cocaine	Opioid	Other
1	DQB1*02:01	0.0485	0.3189	− 0.1136	− 0.1168	0.0734	0.3124
2	DQB1*02:02	0.4550	− 0.0737	0.5776	0.5106	0.2897	− 0.0555
3	DQB1*03:01	− 0.4794	− 0.5949	0.0877	− 0.0089	− 0.3106	− 0.5449
4	DQB1*03:02	0.0602	0.7194	− 0.1617	− 0.2227	0.0008	0.4702
5	DQB1*03:03	0.5058	0.4600	− 0.1491	− 0.1163	0.1403	0.5015
6	DQB1*04:02	0.2381	0.3925	0.0381	− 0.2410	0.1796	0.5645
7	DQB1*05:01	0.6979	0.0146	0.0617	0.2827	0.3145	0.2265
8	DQB1*05:02	− 0.3208	− 0.3109	− 0.3281	− 0.2397	0.0860	− 0.3665
9	DQB1*05:03	− 0.6761	− 0.3728	0.1194	− 0.1661	− 0.2430	− 0.3065
10	DQB1*06:01	− 0.2727	− 0.3880	− 0.2361	− 0.2343	0.2333	− 0.0760
11	DQB1*06:02	0.4739	0.1584	0.0704	0.0447	0.5786	0.5540

**Table 6:** Immunogenetic SUD^HLA^ scores of HLA Class II gene DRB1 alleles.

Index	Allele	Alcohol	Amphetamine	Cannabis	Cocaine	Opioid	Other
1	DRB1*01:01	0.3381	0.4368	− 0.0439	0.2453	0.2690	0.4809
2	DRB1*01:02	0.0311	− 0.0705	− 0.0453	− 0.2248	− 0.0071	− 0.4246
3	DRB1*01:03	0.1734	− 0.3811	0.1762	0.2226	0.1103	0.0270
4	DRB1*03:01	− 0.1627	0.0723	0.6110	0.4131	− 0.0995	− 0.1919
5	DRB1*04:01	0.4399	0.3907	− 0.0047	0.1615	0.2602	0.6879
6	DRB1*04:02	− 0.3202	0.0859	− 0.1751	− 0.2477	− 0.6062	− 0.4802
7	DRB1*04:03	− 0.2809	− 0.1196	− 0.1000	− 0.1755	− 0.1105	− 0.3306
8	DRB1*04:04	− 0.0073	− 0.0377	0.0622	0.0663	0.2373	0.3936
9	DRB1*04:05	− 0.0486	0.0583	0.0840	− 0.1316	− 0.1855	− 0.7013
10	DRB1*04:07	0.0840	− 0.0811	0.4120	0.3822	− 0.4113	− 0.1117
11	DRB1*04:08	0.3753	0.1336	− 0.3736	− 0.3210	0.4244	0.6226
12	DRB1*07:01	0.1296	− 0.3002	0.5768	0.4176	0.0577	− 0.1635
13	DRB1*08:01	0.3465	0.3858	− 0.1830	− 0.2914	0.1224	0.4692
14	DRB1*08:03	0.5360	− 0.0126	− 0.0612	− 0.3081	0.2467	0.1126
15	DRB1*09:01	0.4474	0.4883	− 0.1753	− 0.1110	0.3932	0.5050
16	DRB1*10:01	− 0.4150	− 0.1169	− 0.2121	− 0.0203	− 0.4617	− 0.3588
17	DRB1*11:01	− 0.4620	− 0.3021	− 0.0420	− 0.0480	− 0.2613	− 0.4935
18	DRB1*11:02	− 0.0089	− 0.0250	0.2068	− 0.1179	− 0.0016	− 0.1837
19	DRB1*11:03	− 0.1319	− 0.3426	− 0.0926	− 0.0216	− 0.1947	− 0.5477
20	DRB1*11:04	− 0.4981	− 0.4271	0.0723	0.0189	− 0.2367	− 0.3167
21	DRB1*12:01	0.0904	0.6558	− 0.4334	− 0.2039	0.0282	0.4844
22	DRB1*13:01	0.3829	0.2842	− 0.0326	− 0.2176	0.1739	0.3113
23	DRB1*13:02	0.1608	0.1041	0.3567	0.5473	0.1789	0.3351
24	DRB1*13:03	− 0.1620	− 0.1798	0.2806	0.3138	− 0.3452	− 0.3759
25	DRB1*13:05	0.0882	− 0.3987	− 0.1669	− 0.5244	0.0276	− 0.3023
26	DRB1*14:01	− 0.2713	− 0.4321	− 0.1164	− 0.3365	0.0247	− 0.0626
27	DRB1*15:01	0.3338	0.2307	0.1715	0.3407	0.3021	0.5787
28	DRB1*15:02	− 0.3842	− 0.4118	− 0.2201	− 0.3744	− 0.0598	− 0.2440
29	DRB1*16:01	− 0.4354	− 0.5175	− 0.2030	− 0.0013	− 0.0959	− 0.3339

**Table 7:** Descriptive statistics of the 6 SUD^HLA^ scores.

Substance	Mean	SEM	SD	Median	Minimum	Maximum
Alcohol	0.0287	0.0234	0.2635	0.0388	− 0.6761	0.6979
Amphetamine	− 0.0258	0.0279	0.3141	− 0.0166	− 0.8257	0.7194
Cannabis	− 0.0169	0.0276	0.3111	− 0.0536	− 0.7099	0.7541
Cocaine	− 0.0574	0.0274	0.3088	− 0.0827	− 0.7070	0.6078
Opioid	0.0118	0.0241	0.2716	0.0276	− 0.6062	0.7000
Other	− 0.0157	0.0335	0.3778	− 0.0813	− 0.7013	0.6879

N = 127 scores.

SEM, standard error of the mean; SD, standard deviation.

### Associations between SUD^HLA^ scores

**Figure 2:**
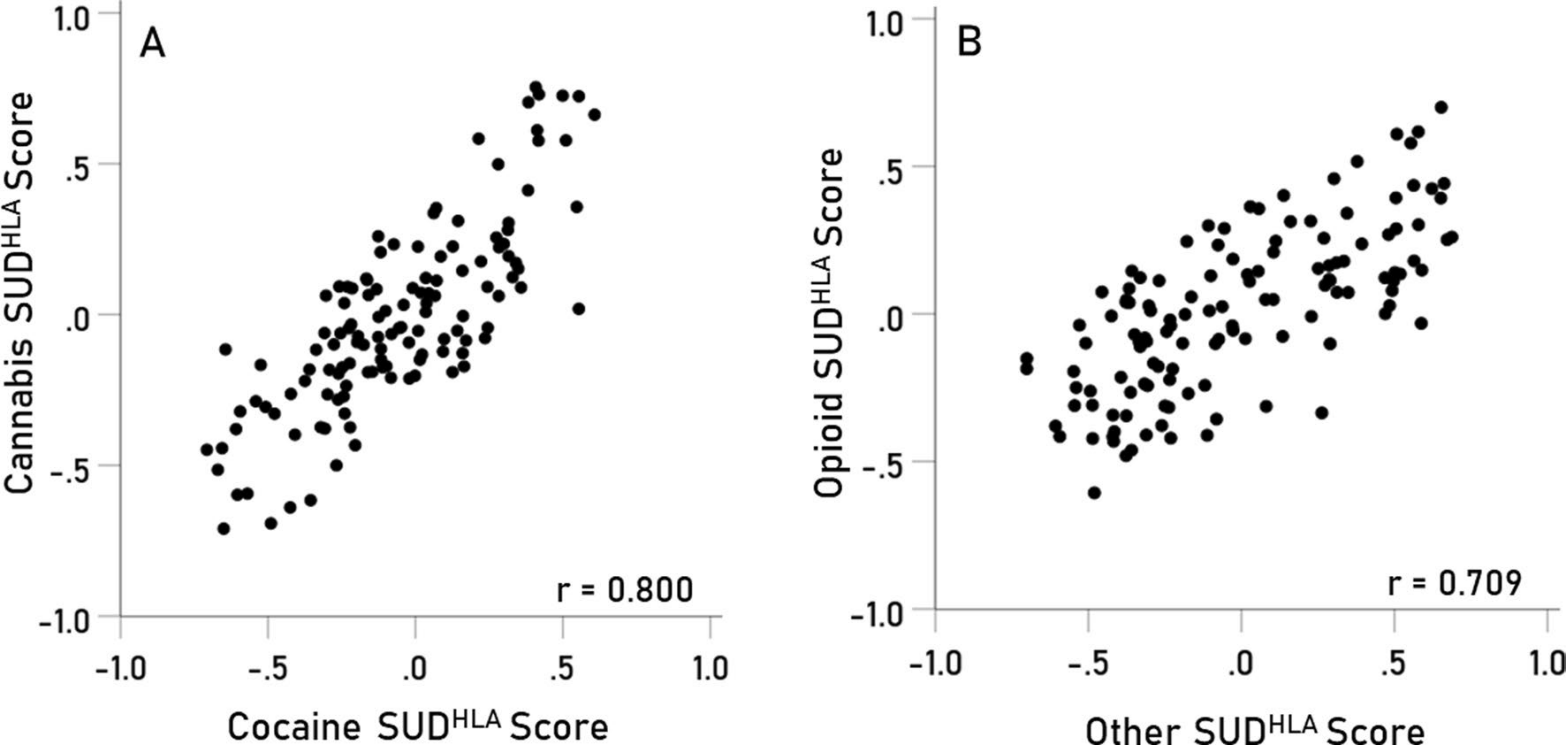
(**A**) cannabis SUD^HLA^ scores are plotted against cocaine use disorder scores (P < 0.001, Table 3). (**B**) opioid use disorder scores are plotted against other substance use disorder (P < 0.001, Table 3).

All 15 pairwise correlations of the SUD^HLA^ scores of the 6 SUDs are given in Table 8. Most notable are the high positive correlations between the cannabis and cocaine scores (Fig. 2A), and between the opioid and “other” scores (Fig. 2B).

**Table 8:** Pearson correlations and associated statistics for the 15 pairwise associations of the SUD^HLA^ scores of the 6 SUDs.

	Pair	$r$	P value	P value Bonferroni	Lower 95% CI	Upper 95% CI
1	**Alcohol—amphetamine**	**0.466**	**P < 0.001**	**P = 4.9 × 10**^**–7**^	**0.318**	**0.593**
2	Alcohol—cannabis	0.154	0.085		− 0.021	0.319
3	Alcohol—cocaine	0.144	0.107		− 0.031	0.310
4	**Alcohol—opioid**	**0.485**	**P < 0.001**	**P = 1.1 × 10**^**–7**^	**0.340**	**0.608**
5	**Alcohol—other**	**0.555**	**P < 0.001**	**P = 1.9 × 10**^**–10**^	**0.422**	**0.665**
6	Amphetamine—cannabis	0.092	0.306		− 0.084	0.262
7	Amphetamine—cocaine	0.144	0.105		− 0.031	0.311
8	Amphetamine—opioid	0.148	0.096		− 0.027	0.314
9	**Amphetamine—other**	**0.570**	**P < 0.001**	**P = 4.2 × 10**^**–11**^	**0.439**	**0.677**
10	**Cannabis—cocaine**	**0.800**	**P < 0.001**	**P = 2.2 × 10**^**–28**^	**0.728**	**0.855**
11	Cannabis—opioid	− 0.152	0.089		− 0.317	0.023
12	Cannabis—other	0.068	0.446		− 0.107	0.240
13	Cocaine—opioid	− 0.070	0.437		− 0.241	0.106
14	Cocaine—other	0.198	0.026		0.024	0.359
15	**Opioid—other**	**0.709**	**P < 0.001**	**P = 1.7 × 10**^**19**^	**0.610**	**0.786**

The highly statically significant correlations are in bold.

*CI* confidence interval.

### Factor analysis of SUD^HLA^ scores

**Figure 3:**
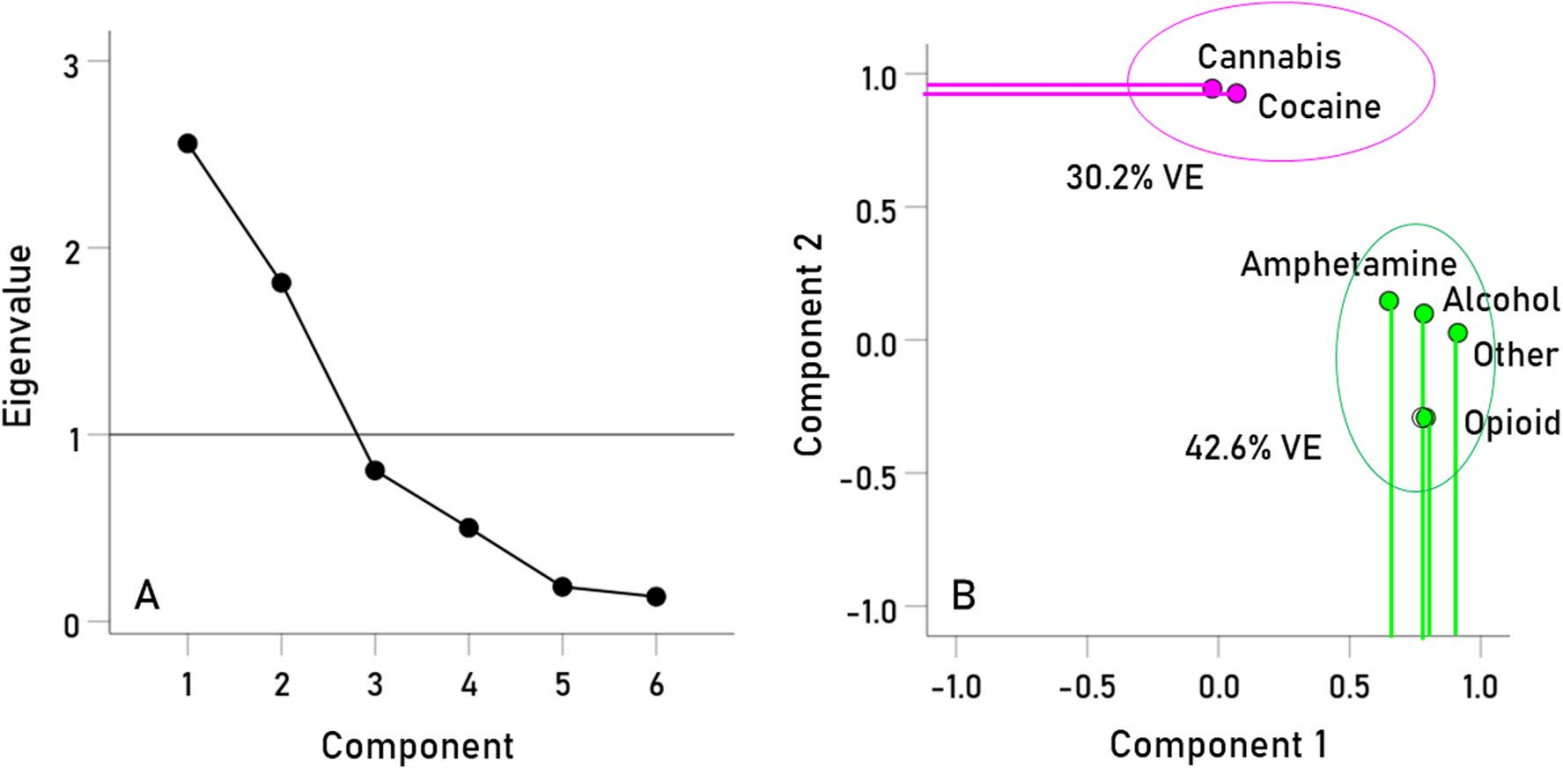
(**A**) scree plot of factor analysis of the 6 SUD^HLA^ scores. (**B**) component plot of factor analysis of the 6 SUD^HLA^ scores. The first component (green cluster) comprises alcohol, amphetamine, opioid, and other SUDs; the second component (magenta cluster) comprises cannabis and cocaine use disorders. See text for details.

The factor analysis yielded 2 components (with eigenvalue > 1) which accounted for 72.9% of the variance (Table 9; Fig. 3A, scree plot). The correlation between the components was very low (r = 0.099). The specific assignment of the 6 SUDs to the 2 factor analysis components was inferred from the factor analysis structure matrix, which provides the correlations between SUD type and factor analysis component (Table 10). It can be seen that alcohol, amphetamine, opioid and “other” SUDs were primarily associated with Component 1, whereas cannabis and cocaine use disorders were primarily associated with Component 2. This is illustrated in the component plot of Fig. 3B, where it can be seen that alcohol, amphetamine, opioid and other disorders project at high values on Component 1, whereas cannabis and cocaine use disorders project highly on Component 2.

**Table 9:** Variance explained by components of SUD^HLA^ scores.

Component	Eigenvalue	Percent of variance explained	Cumulative percent of variance explained
1	2.559	42.646	42.646
2	1.813	30.216	72.862
3	0.808	13.475	
4	0.501	8.358	
5	0.185	3.090	
6	0.133	2.215

**Table 10:** Structure matrix of the 2 factor analysis components.

Substance	Component	
	1	2
Alcohol	**0.792**	0.167
Amphetamine	**0.663**	0.203
Cannabis	0.058	**0.942**
Cocaine	0.150	**0.933**
Opioid	**0.766**	− 0.224
Other	**0.915**	0.106

Values are correlations of a SUD type with a factor analysis component. The higher of the two SUD/component values are in bold.

### Application to individuals: assessment of SUD risk based on the individual’s whole HLA profile

Since each individual carries a total of 12 HLA alleles (2 per 6 classical HLA genes), we used the average (τ, Eq. 2) of the 12 SUD^HLA^ scores of an individual as an estimate of the risk of that individual for a particular SUD. In order to be able to interpret this risk measure, we standardized τ with respect to a large simulated population by generating, for each SUD, a large sample of expected τ* values (N = 1,000,000) using a bootstrap procedure, where τ* was the sum of 12 randomly selected SUD^HLA^ scores (2 per gene). The resulting frequency distributions of τ* were unimodal, approximating a normal distribution; descriptive statistics of these distributions of the 6 SUD τ* values are given in Table 11. The Pearson correlations between the 6 SUD τ* distributions were very similar to those of SUD^HLA^ (Table 8) and are given in Table 12. Similarly, the same factor analysis applied to the τ* distributions yielded the same number of components (Table 13) and component structure matrix (Table 14) as the distributions of SUD^HLA^ scores (Tables 9 and 10).

**Table 11:** Descriptive statistics of the 6 SUD^HLA^ τ*scores (N = 1,000,000 per row).

Substance	Mean	SEM	SD	Median	Minimum	Maximum
Alcohol	0.01282	0.000088	0.088216	0.01314	− 0.399	0.380
Amphetamine	− 0.02855	0.000090	0.089759	− 0.03000	− 0.388	0.409
Cannabis	− 0.01130	0.000072	0.072384	− 0.00872	− 0.282	0.412
Cocaine	− 0.01909	0.000090	0.079970	− 0.02022	− 0.338	0.374
Opioid	− 0.00765	0.000073	0.073481	− 0.00606	− 0.378	0.312
Other	− 0.02111	0.000118	0.117623	− 0.02283	− 0.509	0.486

SEM, standard error of the mean; SD, standard deviation.

**Table 12:** Pearson correlations and associated statistics for the 15 pairwise associations of the SUD^HLA^ τ* scores of the 6 SUDs.

Pair	$r$	P value	Lower 95% CI	Upper 95% CI
**Alcohol—amphetamine**	**0.657**	**P < 0.001**	**0.656**	**0.658**
Alcohol—cannabis	0.032	0.085	0.030	0.034
Alcohol—cocaine	0.071	0.107	0.069	0.073
**Alcohol—opioid**	**0.768**	**P < 0.001**	**0.767**	**0.769**
**Alcohol—other**	**0.759**	**P < 0.001**	**0.759**	**0.760**
Amphetamine—cannabis	− 0.161	0.306	− 0.163	− 0.159
Amphetamine—cocaine	0.063	0.105	0.061	0.065
Amphetamine—opioid	0.431	0.096	0.430	0.433
**Amphetamine—other**	**0.699**	**P < 0.001**	**0.698**	**0.700**
**Cannabis—cocaine**	**0.790**	**P < 0.001**	**0.790**	**0.791**
Cannabis—opioid	− 0.109	0.089	− 0.111	− 0.107
Cannabis—other	− 0.142	0.446	− 0.144	− 0.140
Cocaine—opioid	− 0.027	0.437	− 0.029	− 0.025
Cocaine—other	0.130	0.026	0.128	0.132
**Opioid—other**	**0.803**	**P < 0.001**	**0.802**	**0.803**

*CI* confidence interval.

**Table 13:** Total variance explained by components of SUD^HLA^ τ* scores .

Component	Eigenvalue	Percent of variance explained	Cumulative percent of variance explained
1	3.089	51.477	51.477
2	1.799	29.991	81.469
3	0.595	9.923	
4	0.322	5.368	
5	0.113	1.889	
6	0.081	1.351

**Table 14:** Structure matrix of the 2-component FA for SUD types.

Substance	Component	
	1	2
Alcohol	**0.908**	0.064
Amphetamine	**0.788**	− 0.053
Cannabis	− 0.116	**0.948**
Cocaine	0.071	**0.944**
Opioid	**0.862**	− 0.074
Other	**0.938**	− 0.007

Values are correlations of a SUD^HLA^ τ* for each SUD type with a FA component. The higher of the two SUD/component values are in bold.

**Figure 4:**
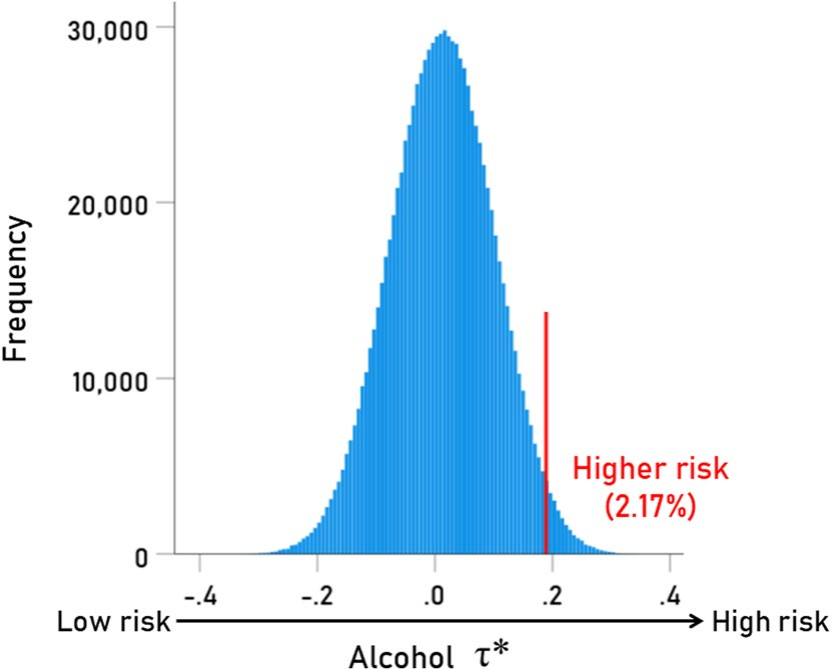
Frequency distribution of τ* for Alcohol (N = 1,000,000). See text for details.

The τ* distribution for alcohol is shown in Fig. 4. The red line is at the level of mean + 2 SD, thus providing an estimated threshold of excessive alcohol SUD risk, along the rationale of using T score in estimating risk related to bone density. We employed a similar approach here and used the z-score of the τ* distribution as a continuously varying risk score. The relevant computations are shown in Table 15, where T thresholds for excessive risk (> mean + 2SD) are given for each SUD. For a given individual, the only information needed to compute their T score is the set of the 12 HLA alleles the individual carries: then, using Tables 1, 2, 3, 4, 5, 6, the average τ score is calculated and its z-score (for a particular SUD) is computed and referred to the threshold(s) in Table 15 for assessment of the risk. As an applied exercise, we calculated the T scores for the best (lowest SUD^HLA^) and worst (highest SUD^HLA^) cases by averaging the 2 smallest (for the former case) and the 2 largest (for the latter case) SUD^HLA^ scores from each one of the 6 genes, yielding the τ_min_ and τ_max_, respectively. The relevant data, τ, and T values are given in Tables 16 and 17, for lowest and highest risk assessments.

**Table 15:** T-score calculation for estimated individual SUD risk.

SUD	$\mathrm{Mean}$ SUD τ*	SD	T-score =	Risk	Higher risk threshold (mean + 2SD)
Alcohol	0.01282	0.088216	$\frac{\tau -Mean}{SD}$	$T<-2 \;\;\mathrm{Lower}$ $2\ge T\ge -2 \;\; \mathrm{Average}$ $T>2 \;\; \mathrm{High}$er	τ > 0.18925 (2.17%)
Amphetamine	− 0.02855	0.089759			τ > 0.15097 (2.50%)
Cannabis	0.01130	0.072384			τ > 0.15607 (2.81%)
Cocaine	− 0.01893	0.079970			τ > 0.14101 (2.49%)
Opioid	− 0.00750	0.073481			τ > 0.13946 (1.90%)
Other	− 0.02111	0.117623			τ > 0.21414 (2.44%)

N = 1,000,000 per SUD*. See text for details.

**Table 16:** Lowest risk HLA genotypes for the 6 SUD studied.

	Allele	Alcohol	Amphetamine	Cannabis	Cocaine	Opioid	Other
1	A1	A*11:01	A*01:01	A*11:01	A*02:01	A*29:02	A*02:05
2	A2	A*30:01	A*32:01	A*30:02	A*30:02	A*33:01	A*29:02
3	B1	B*13:02	B*15:18	B*27:02	B*39:06	B*14:02	B*49:01
4	B2	B*37:01	B*57:01	B*40:02	B*44:05	B*39:01	B*51:01
5	C1	C*07:01	C*07:04	C*04:01	C*01:02	C*06:02	C*12:03
6	C2	C*12:02	C*14:02	C*07:04	C*04:01	C*15:02	C*14:02
7	DPB1	DPB1*02:01	DPB1*04:02	DPB1*03:01	DPB1*03:01	DPB1*02:01	DPB1*02:01
8	DPB2	DPB1*09:01	DPB1*13:01	DPB1*04:02	DPB1*04:02	DPB1*03:01	DPB1*17:01
9	DQB1	DQB1*03:01	DQB1*03:01	DQB1*05:02	DQB1*04:02	DQB1*03:01	DQB1*03:01
10	DQB2	DQB1*05:03	DQB1*06:01	DQB1*06:01	DQB1*05:02	DQB1*06:09	DQB1*06:09
11	DRB1	DRB1*11:01	DRB1*14:01	DRB1*04:08	DRB1*13:05	DRB1*04:02	DRB1*04:05
12	DRB2	DRB1*11:04	DRB1*16:01	DRB1*12:01	DRB1*15:02	DRB1*10:01	DRB1*11:03
	τ	− 0.3750	− 0.5176	− 0.4582	− 0.4505	− 0.4239	− 0.5399
	T	− 4.3963	− 5.4485	− 6.4862	− 5.3966	− 5.6668	− 4.4106

The 2 alleles for each gene/SUD combination have the 2 most negative HLA-SUD scores (Table 2). τ is the average of the scores of the 12 alleles in a column. T is the z-score of τ.

**Table 17:** Highest risk HLA genotypes for the 6 SUD studied.

	Allele	Alcohol	Amphetamine	Cannabis	Cocaine	Opioid	Other
1	A1	A*02:01	A*03:01	A*23:01	A*24:02	A*03:01	A*26:01
2	A2	A*68:02	A*31:01	A*24:02	A*29:02	A*68:02	A*68:01
3	B1	B*14:01	B*14:01	B*44:03	B*44:03	B*52:01	B*40:01
4	B2	B*39:06	B*37:01	B*55:01	B*55:01	B*56:01	B*56:01
5	C1	C*03:03	C*03:03	C*05:01	C*05:01	C*01:02	C*01:02
6	C2	C*05:01	C*07:02	C*16:01	C*16:01	C*03:03	C*03:03
7	DPB1	DPB1*04:01	DPB1*03:01	DPB1*06:01	DPB1*06:01	DPB1*04:01	DPB1*01:01
8	DPB2	DPB1*06:01	DPB1*09:01	DPB1*11:01	DPB1*11:01	DPB1*04:02	DPB1*04:01
9	DQB1	DQB1*05:01	DQB1*03:02	DQB1*02:02	DQB1*02:02	DQB1*05:01	DQB1*04:02
10	DQB2	DQB1*06:03	DQB1*03:03	DQB1*05:03	DQB1*06:04	DQB1*06:02	DQB1*06:02
11	DRB1	DRB1*08:03	DRB1*09:01	DRB1*03:01	DRB1*07:01	DRB1*04:08	DRB1*04:01
12	DRB2	DRB1*09:01	DRB1*12:01	DRB1*07:01	DRB1*13:02	DRB1*09:01	DRB1*04:08
	τ	0.4237	0.5135	0.6056	0.4583	0.4713	0.6051
	T	4.6577	6.0389	8.2104	5.9676	6.5160	5.3239

The 2 alleles for each gene/SUD combination have the 2 most positive HLA-SUD scores (Table 2). τ is the average of the scores of the 12 alleles in a column (Eq. 2). T is the z-score of τ (Table 10).

## Discussion

Here we used an epidemiological approach to evaluate the immunogenetic profiles of 6 SUDs and their associations and to estimate individual SUD risk. We documented robust immunogenetic associations between SUDs at both the population and individual level characterized by two groupings—one comprised solely of cannabis and cocaine dependence and the other of alcohol, amphetamine, opioid, and other dependence. These findings, which provide novel evidence of immunogenetic associations with SUDs, are discussed below.

Relatively few studies have focused on HLA-SUD associations, and most previous HLA-SUD association studies have been limited to alcohol use disorders. Several studies in 1980s documented HLA associations with alcohol use disorders and sequelae, although findings across studies were inconsistent^14^ and that line of research subsequently dwindled; however, recent advances in genetic association studies have renewed interest in the role of HLA in alcohol use disorders. To that end, a recent candidate gene association study identified several single nucleotide polymorphisms (SNPs) in the HLA-DRA gene that were associated with alcohol dependence^12^, and epigenetic changes of several genes related to inflammation and immune system regulation including HLA have been reported among those with alcohol use disorders^19^. Our findings suggest that HLA-SUD associations extend beyond alcohol to other addictive substances and highlight immunogenetic groupings among SUDs.

We found evidence of two distinct groups of SUDs based on their immunogenetic profiles. Previous research evaluating genetic and environmental risk for SUDs in twins identified two genetic factors for SUDs that, remarkably, correspond with findings from the present study^20^. Specifically, similar to our findings, Kendler et al.^20^ found that cocaine and cannabis loaded onto 1 genetic factor, whereas other SUDs (licit substances including alcohol, nicotine, and caffeine dependence) loaded onto a separate, albeit highly intercorrelated, genetic factor. We did not evaluate licit SUDs other than alcohol in our study although previous research indicates caffeine and nicotine are largely influenced by unique genetic factors^20^. Two large twin studies of illicit drugs found predominantly common genetic risk shared across illicit SUDs^21,22^, with modest specific genetic influences on risk for some drugs^22^. Our findings, which utilize a different approach based on population immunogenetics, extend the literature by evaluating the influence of HLA genes on population and individual risk for SUDs, and document that genes involved in the immune response to foreign antigens are associated with two clusters of SUDs based on immunogenetic profiles.

Cannabis and cocaine dependence formed one HLA-based SUD group that was distinguished from the other grouping containing all four of the other SUDs. It is notable that cannabis and cocaine SUD^HLA^ profiles were very highly correlated both at the population level and for individual SUD risk. Their correspondence was further reflected in analyses identifying the 2 alleles (out of 127) associated with the highest risk of each SUD. For cannabis and cocaine, the high risk alleles were virtually identical (9 out of 12 alleles) and minimally overlapped with high risk HLA alleles for SUDs in the other group (Table 12). In fact, in some cases the genes associated with the highest risk for an SUD from one group were associated with the lowest risk for an SUD in the other group as exemplified by A*02:01 conferring high risk for alcohol and low risk for cocaine (Table 11). Prior studies reviewed elsewhere^23^ have highlighted links between cannabis and cocaine including similar neuropharmacological actions of cannabinoids and cocaine, and endocannabinoid system involvement in cocaine addiction. In contrast, cannabidiol has been shown to inhibit the reward-facilitating effect of opioids and other substances that were part of the second cluster in the present study^24,25^. Beyond differences in brain reward effects, the separate clustering of cocaine and cannabis from the other SUDs investigated here documents immunogenetic differences between the two clusters of SUDs. Similar to the cannabis-cocaine grouping, the finding that alcohol, amphetamines, opioids, and “Other” dependencies clustered together suggests common HLA associations amongst those SUDs that differ from the cannabis-cocaine cluster. Indeed, the SUD^HLA^ correlations among alcohol, amphetamine, opioid, and “Other” were considerably stronger than their associations with cannabis or cocaine. Taken together, the present findings highlight similarities and difference in the immunogenetic profiles of SUDs. The HLA-SUD associations documented here are particularly interesting in light of research on immunotherapies for treatment of addictions including several Phase I and II clinical trials evaluating the effectiveness of anti-addiction vaccines and antibodies aimed at preventing drugs from reaching the brain and activating reward centers^26^.

To our knowledge, this is the first study to evaluate immunogenetic profiles of SUDs and their associations. The findings of this immunogenetic epidemiological study provide novel insights regarding HLA-SUD associations; however, the findings must be considered within the context of study limitations. First, this was an epidemiological study. We utilized the population level SUD^HLA^ scores to estimate individual risk although future studies are warranted to determine whether the individual risk estimates are corroborated in vivo. Second, the current study focused on Continental Western Europe. Since geographic and ethnic variation in HLA are well-established^27,28^ and SUD prevalence varies globally^1^, the HLA-SUDs associations identified here may vary in other regions. An additional consideration involves reporting of illicit substances. Some individuals may be hesitant to disclose problematic substance use which may impact estimates; however, potential reporting biases are somewhat mitigated by the fact that population estimates of SUDs used in the present analyses were obtained from the Global Burden of Disease study which is the most comprehensive epidemiological study of diseases including SUDs. Finally, many other genetic and environmental factors not investigated here contribute to SUDs; how those factors interact with HLA to influence SUD prevalence remains to be investigated.

## Materials and methods

### Epidemiological data

#### Prevalence of substance use disorders

The population prevalence of alcohol use disorder, amphetamine use disorder, cannabis use disorder, cocaine use disorder, opioid use disorder, and other drug use disorders in 2019 was computed for each of the following 14 countries in Continental Western Europe (CWE): Austria, Belgium, Denmark, Finland, France, Germany, Greece, Italy, Netherlands, Portugal, Norway, Spain, Sweden, and Switzerland. Specifically, the total number of people with each SUD in each of the 14 CWE countries was identified from the Global Health Data Exchange^29^, a publicly available catalog of data from the Global Burden of Disease study, the most comprehensive worldwide epidemiological study of more than 350 diseases. The number of people with each SUD in each country was divided by the total population of each country in 2016^30^ and expressed as a percentage. We have previously shown that life expectancy for these countries is virtually identical^31^; therefore, life expectancy was not included in the current analyses.

#### HLA

The frequencies of all reported HLA alleles of classical genes of Class I (A, B, C) and Class II (DPB1, DQB1, DRB1) for each of the 14 CWE countries were retrieved from the website allelefrequencies.net (Estimation of Global Allele Frequencies)^32,33^ on October 20, 2020. As we reported previously^31^, there were 844 distinct alleles, i.e. alleles that occurred in at least one country. Of those, 127 alleles occurred in 9 or more countries and were used in further analyses. This criterion is somewhat arbitrary but reasonable; it was partially validated in a previous study^34^.

### Data analysis

#### HLA-SUD profiles

HLA-SUD profiles for each SUD disorder above were derived by computing the covariance between the nonparametric normal scores^35^ of the prevalence of a SUD and those of the population frequency of an allele, comprising 69 HLA Class I and 58 Class II alleles, for a total of 127 alleles. The covariance can be negative or positive, indicating a negative or positive association, respectively. The equation for the HLA-SUD score is:

1 $${\text{SUD}}^{{{\text{HLA}}}} \;{\text{score}} = \frac{1}{N - 1}\sum\limits_{i}^{i = 1,N} {\left( {f_{i} - \overline{f}} \right)\left( {p_{i} - \overline{p}} \right)}$$

where ${f}_{i}, {p}_{i}$ denote the normal scores of allele frequency and SUD prevalence for the *i*th country, respectively, and $\overline{f },\overline{p }$ are their means. Thus a SUD^HLA^ profile is a vector with 127 HLA-SUD scores. It should be noted that covariance is a descriptive measure of interdependence not subject to formal statistical testing and one that has been used routinely for many years routinely and successfully in other fields, including evolutionary biology^36^ and finance^37^.

Standard statistical methods were used to analyze the HLA-SUD scores, including parametric univariate (mean, standard deviation, etc.), bivariate (Pearson correlation), multivariate (factor analysis), and permutations-based statistics.

#### Random permutations test for assessing the statistical significance of the HLA-SUD profiles

In this analysis, we tested the null hypothesis that the HLA-SUD profiles may be due to chance by performing a permutation test, where the pairing of allele frequencies and SUD prevalences was randomly scrambled. More specifically, let $H$ be HLA-SUD profile for a specific SUD, and let *H*′ be the profile obtained after random pairing of pairing of alleles and countries. If the profiles are identical, the sum *S* of the absolute paired differences between them ($H$,*H*′) would be zero. We carried out this procedure 1,000,000 times for each one of the 6 HLA-SUD profiles and counted the number of times *M* for which the sum *S* was equal to zero, indicating that the randomly obtained profile would be the same as the observed one. Then, the ratio $w=\frac{M}{\mathrm{1,000,000}}$ is the probability that the observed profile $H$ could be due to chance. In a relaxed variation of the test, we computed the sum of the absolute differences between the ranked profiles.

#### Factor analysis

A factor analysis (FA) were performed to identify potential groupings (“components”) of SUD_HLA_ scores. The method of principal components was used for extraction and the method of direct oblimin (delta = 0) with Kaiser normalization was used for factor rotation.

#### Application to individuals

Since every individual carries *k* = 12 classical HLA alleles (2 of each 3 HLA Class I and 3 Class II genes), average SUD^HLA^ scores were calculated:

2 $$\uptau =\frac{1}{12}\sum_{k}^{k=\mathrm{1,12}}{\mathrm{SUD}}^{\mathrm{HLA}}(k)$$

We obtained expected estimates of τ using a bootstrap procedure^38^, as follows. For each HLA gene and SUD, two SUD^HLA^ scores were drawn randomly (with replacement) from the pool of available alleles and were averaged to yield bootstrap values of τ* for a simulated “individual”. The procedure was repeated 1 million times for a total of 1,000,000 τ* values which were used for further analyses. The same random seed was used for each draw of the 12 SUD^HLA^ values, such that SUD^HLA^ values for all 6 SUDs referred to the same set of alleles, thus allowing for an assessment of associations between τ* distributions.

#### Implementation of analysis procedures

The IBM-SPSS statistical package (version 27) was used for implementing standard statistical analyses. All P values reported are 2-sided. The permutation test and bootstrap procedure was implemented using FORTRAN (Geany, version 1.38, built on or after 2021–10-09) and 64-bit Mersenne Twister random number generator with a large random double-precision odd seed.

## Data Availability

All data used were retrieved from freely accessible websites^29–33^, and, as such, are publicly and freely available.
